# Effect of helmet design on impact performance of industrial safety helmets

**DOI:** 10.1016/j.heliyon.2022.e09962

**Published:** 2022-07-16

**Authors:** Michael Bottlang, Gina DiGiacomo, Stanley Tsai, Steven Madey

**Affiliations:** Biomechanics Laboratory, Legacy Research Institute, Portland, OR, 97232, USA

**Keywords:** Construction, Safety helmet, Hard hat, Brain injury, Concussion, Impact testing

## Abstract

**Background:**

Comparative studies of different helmet designs are essential to determine differences in helmet performance. The present study comparatively evaluated the impact performance of hardhat helmets, climbing-style safety helmets, and helmets with novel rotation-damping technologies to determine if advanced designs deliver improved protection.

**Methods:**

Six helmet designs from three categories of safety helmets were tested: two traditional hardhat helmets (HH Type I, HH Type II), two climbing-style helmets (CS Web, CS Foam), and two helmets with dedicated rotation-damping technologies (MIPS, CEL). Helmets were first evaluated in impacts of 31 J energy representing a falling object according to standard Z89.1–2014. Subsequently, helmets were evaluated in impacts representing a fall by dropping a helmeted head-neck surrogate at 275 J impact energy. The resulting head kinematics were used to calculate the probability of sustaining a head or brain injury.

**Results:**

Crown impacts representative of a falling object resulted in linear acceleration of less than 50 g in all six helmet models. Compared to crown impacts, front, side and rear impacts caused a several-fold increase in head acceleration in all helmets except HH Type II and CEL helmets. For impacts representative of falls, all helmets except the CEL helmet exhibited significantly increased head accelerations and an increased brain injury probability compared to the traditional HH Type I hardhat. Neck compression was 35%–90% higher in the two climbing-style helmets and 80% higher in MIPS helmets compared to the traditional HH type I hardhat.

**Discussion:**

Contemporary helmets do not necessarily deliver improved protection from impacts and falls compared to traditional hardhat helmets.

## 1 Introduction

Work-related traumatic brain injury (TBI) accounts for 18% of the estimated 1.7 million TBIs that occur in the Unites States annually [1, 2]. It is among the most serious and disruptive occupational injuries, as those surviving TBI can face long-term cognitive, psychological and emotional impairments [3, 4]. The economic burden of treatment for severe TBI ranges from $600,000 to $1,8 million per case, not including the value of lost productivity [5, 6]. The construction industry faces the highest number of occupational TBIs of any industry in the US, accounting for 25% of all work-related TBIs [7]. Falls are responsible for 68% of all work-related TBI cases in the construction industry, while falling objects cause only 12% of work-related TBI [8]. Outside of construction, falls remain the leading cause of work-related TBI, accounting for 64% of all cases [9]. A recent analysis of workers’ compensation systems in Sweden and Germany similarly found that the most frequent events leading to work-related TBI were falls [10]. The study concluded that helmet testing standards should evaluate protection against fall-induced TBI as well as skull fractures from falling objects. Among work-related TBIs sustained from falls, 51% of the falls occur from a height of less than 6 ft, and 40% of these falls are ground level tripping events [8]. A 2021 systematic review found that despite a decrease in overall work-related injury claims, the proportion of claims from work-related TBIs have increased [2]. This suggests that advancing preventative safety technology remains an urgent matter to address the costly and debilitating TBI epidemic among the work force.

Helmets are the most effective intervention to reduce the incidence and severity of work-related head injury [11]. Despite being the principal preventative measure, today's most frequently used hardhats remain highly similar in design to their predecessors from 70 year ago [12]. Two reasons may attribute to this apparent lack of technological progress. First, helmets are designed to meet national standards that prescribe minimum performance criteria but are not intended to assess or drive technological advances. Second, while independent research and testing is driving technology implementation to improve performance of sports helmets [13, 14, 15, 16, 17], comparative testing on the effectiveness of industrial safety helmets remains rare at best.

The standard for industrial head protection Z89.1–2014 of the American National Standard Institute (ANSI) specifies impact protection requirements for two helmet types, Type I and Type II. Type I helmets are only tested for impacts onto the helmet apex. Impact tests employ a falling impactor with a sharp tip to assess penetration resistance or a drop onto a hemispherical impactor to assess force transmission. Type I helmets therefore do not account for the fact that only a quarter to a third of impacts occur on the helmet crown, while 52%–62% of impacts occur to the helmet front and sides [18]. Type II helmets must in addition provide impact energy attenuation and penetration resistance for off-center impacts to the helmet front, back, and sides. Impact energy attenuation is measured by a vertical drop of a helmeted headform onto a hemispherical anvil at 3.5 m/s, corresponding to an 0.6 m free-fall height. This test setup simulates an impact from a falling object. However, the drop height of 0.6 m falls short of the vertical drop height of the head in a ground level fall, and it does not represent falls from an elevated height. Furthermore, the corresponding impact energy of 31 J for testing of industrial hard hats is several times lower than impact energies used for standard testing of recreational helmets, such as bicycle helmets (58 J–96 J) [19], equestrian helmets (88 J) [20], and mountaineering helmets (98 J) [21] that simulate falls. Therefore, while Type II helmets provide added protection from off-center impacts, they are not tested at energy levels representative of realistic falls. Most importantly, neither Type I nor Type II helmets are tested for their ability to mitigate rotational forces caused by real-world falls which are known to induce both linear and rotational accelerations to the head. A large body of research has demonstrated that rotational forces, as measured in terms of rotational acceleration and rotational velocity, are the principal cause of concussions and TBI [22, 23, 24, 25, 26, 27].

Hardhats with an injection-molded polymer shell and a harness suspension were introduced in the 1950s. Seven decades later, they remain today's most widely used hardhat configuration in a largely original design. Most hardhat helmets that meet ANSI Type I requirements use a 4 or 6 point harness that suspends a polymer shell at a set distance of about 3–5 cm over the head apex. Impact absorption relies on deformation of the helmet apex within the air space between the shell and the harness suspension. In side impacts, these helmets provide surprisingly little protection since the harness does not effectively prevent the shell from contacting the head [18]. Type II hardhats employ a liner of expanded polystyrene (EPS) foam on the inside of the helmet shell, in addition to the harness suspension. For front, back and side impacts, this EPS liner provides the primary means of impact force mitigation. More recently, climbing-style safety helmets have been introduced that combine the design of recreational hard-shell sports helmets with the requirements of safety helmets. Similar to hardhats, they have an injection-molded polymer shell but no brim. They have either a harness suspension, an EPS lined shell, or a combination of both. Climbing-style safety helmets are typically fitted with a retention system and a chin strap to retain the helmet on the head during a fall. While they are perceived to provide improved protection compared to standard hardhats, there remains a lack of research quantifying this perceived improvement in impact performance. Most recently, helmet technologies have been introduced that are specifically designed to mitigate rotational forces known to cause concussions and TBI. In 2021, the first climbing-style safety helmet with Multidirectional Impact Protection System (MIPS) technology was introduced. This helmet, the Nexus Extreme MIPS, has a low friction layer that allows the head to move inside the helmet to redirect harmful rotational forces that otherwise are transferred to the head [16, 17, 28]. Similarly, WaveCel technology has been introduced in bicycle and snow sport helmets to mitigate rotational forces by providing a cellular energy mitigation system. MIPS and WaveCel technologies have been shown to significantly reduce rotational head acceleration and the corresponding TBI risk in bicycle [14] and snow [15] helmets, but they have not been tested in construction helmets.

The present study comparatively evaluated the impact performance of Type I and Type II hardhats, climbing-style safety helmets, and safety helmets equipped with dedicated rotation-damping technologies. Impact attenuation of helmets was tested according to ANSI standard Z89.1–2014 at 31 J for crown, front, side, and rear impacts. Additionally, helmets were tested in oblique impacts representative of a fall to assess mitigation of rotational forces in a more realistic impact scenario that accounts for linear and rotational head acceleration and head rotational velocity. Results were used to test the hypothesis that compared to a standard Type I hardhat helmet, advanced helmet designs deliver improved mitigation of linear and rotational impact forces to provide a higher level of protection from concussion and head injury.

## 2 Methods

### 2.1 Helmets

**Figure 1:**
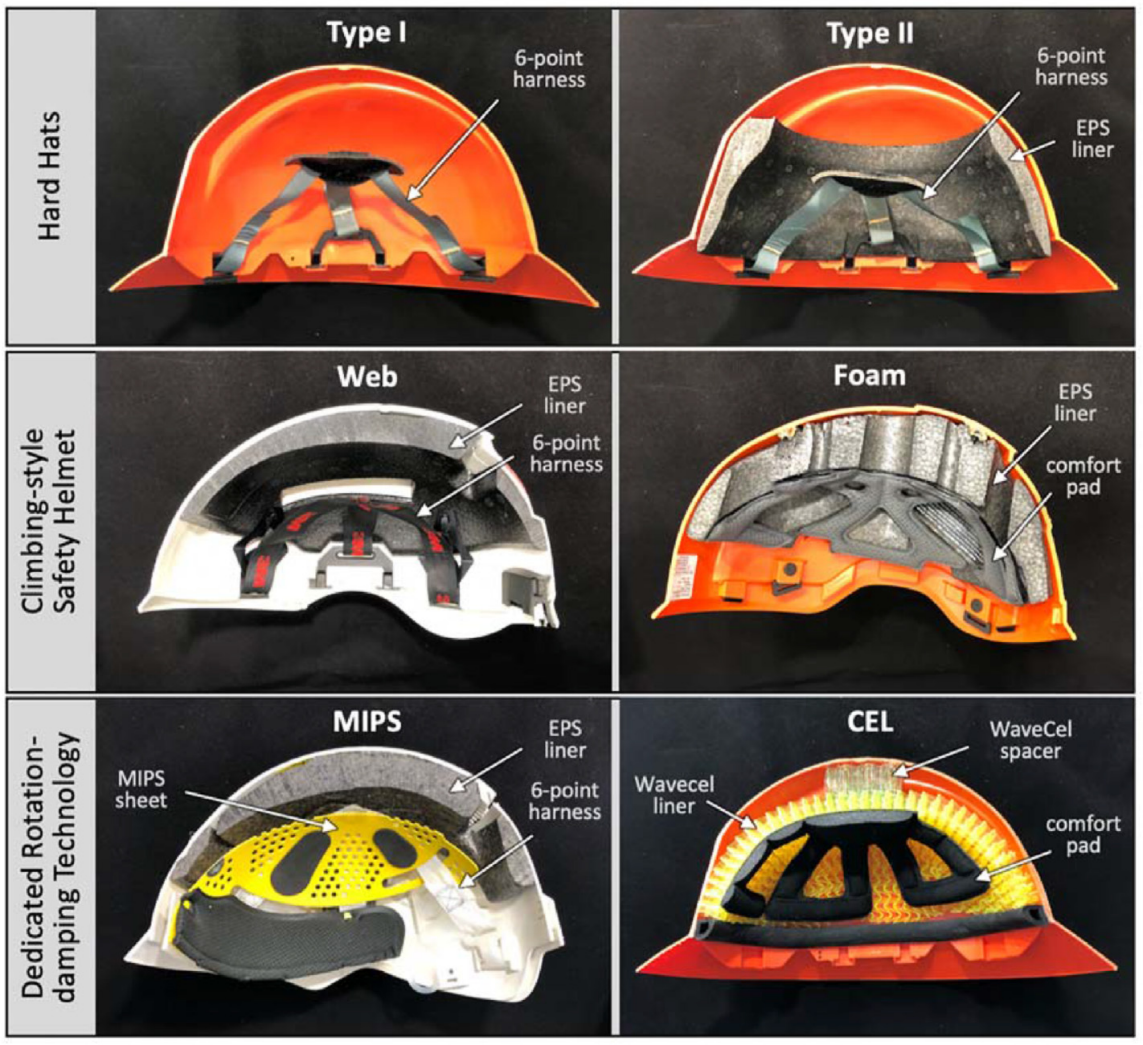
Cross-sectional view of the six industrial safety helmets and their components for impact mitigation.

Six industrial safety helmets comprising three categories were evaluated: two full-brim hardhats, two climbing-style safety helmets, and two helmets equipped with dedicated rotation-damping technologies (Figure 1).

Two standard full-brim hardhats were selected from Honeywell (Charlotte, North Carolina), since they offer the same helmet model (Everest) in a Type I version (model A49R) and in a Type II version (model A119R). Both version had the same shell made from High Density Polyethylene (HDPE). The Type I version (group HH Type I) had a 6-point harness suspension and was used for representation of baseline performance of a standard hardhat. The Type II version (group HH Type II) had the same 6-point harness suspension. In addition, it featured a 13–27 mm thick EPS liner of 95 gpl density that lined the front, sides, and rear, but not the crown of the shell.

Two climbing-style safety helmets were selected. The first model (group CS WEB) had a 6-point harness webbing and a 9–20 mm thick EPS liner of 81 gpl density that partially lined the helmet shell (SecureFit X5000, 3M, St Paul, Minnesota). The second climbing style model (group CS Foam) had a full thickness EPS foam liner of 29 gpl density, ranging in thickness from 12-40 mm (Zenith X, Kask, Chiuduno, Italy). The foam liner was covered by a comfort pad. Both climbing-style safety helmets complied with ANSI standard Z89.1–2014 Type I.

Two helmets with a dedicated rotation-damping technology were tested. One model (group MIPS) was a climbing style helmet claimed to be the first safety helmet with a MIPS cradle (Centurion Nexus Extreme MIPS, City, State). MIPS is a low friction layer designed to reduce the transfer of rotational motion from oblique impacts to the head. The MIPS low friction layer consists of a 1 mm thick plastic sheet that is elastically suspended inside a 6-point harness to allow for 10–15mm slip motion between the helmet and head. In addition to MIPS and the harness suspension, this helmet also had an 8–27 mm thick EPS liner of 53 gpl (grams/liter) density inside the ABS shell. It complied with the European standard EN 12492:2012 for Mountaineers’ safety helmets.

To test the effects of WaveCel rotation-damping technology (WaveCel, Wilsonville, Oregon) in industrial safety helmets, prototype hardhats with a WaveCel liner were manufactured (group CEL). Starting with standard hardhats identical to group HH Type I (Everest A49R), the 6-point harness suspension was replaced by a 15mm thick WaveCel liner, which is a collapsible cellular structure for mitigation of linear and rotational forces. For linear impact forces, each cell has a transverse crease to initiate organized cell buckling similar to a crumple zone. To mitigate the rotational moment during impact, cells can fold in shear direction and the structure can elastically deform in-plane to serve as a rotational suspension between the head and the helmet shell. Since the WaveCel liner was only 15 mm thick, a 22 mm thick WaveCel puck of 56 mm diameter was added to the crown to span the space between the WaveCel liner and the helmet shell. On the helmet inside, a standard open-cell comfort pad was added, similar to climbing style helmets. Additional technical information describing all six helmet models is summarized in Table 1.

**Table 1:** Summary of technical information for the six helmet models selected for testing.

Category	Hard Hat		Climbing-style Safety Helmet		Dedicated Rotation-Damping Technolology	
Group	HH Type I (Baseline)	HH Type II	CS Web	CS Foam	MIPS	CEL (prototype)
Model	Honeywell Everest A49R	Honeywell Everest A119R	3M SecureFit X5000	Kask Zenith X	Centurion Nexus Extreme MIPS	Everest Shell + WaveCel liner
Impact Rating	Type I	Type II	Type I	Type I	Type I	n/a
Style	full-brim hard hat	full-brim hard hat	brimless, climbing style	brimless, climbing style	brimless, climbing style	full-brim hard hat
Image						
Heigh Offset∗	5.5 cm	6.1 cm	5.3 cm	4.7 cm	5.7 cm	5.4 cm
Weight [g]	474 g	535 g	470 g	472 g	534 g	568 g
Outer Shell	HDPE	HDPE	ABS	ABS	ABS	HDPE
Shell Thickness	2.7 mm	2.7 mm	4 mm (crown), 2 mm (side)	2.8 mm	3 mm (crown), 2 mm (side)	2.7 mm
Impact Liner∗∗	non	EPS foam	EPS foam	EPS foam	MIPS sheet + EPS foam	WaveCel
EPS density	n/a	95 ± 3 gpl	81 ± 4 gpl	29 ± 5 gpl	53 ± 6 gpl	n/a
EPS Compr. Modulus	n/a	16.3 kPa	14.8 kPa	6.2 kPa	10.1 kPa	n/a
Liner thickness: crown	n/a	n/a	20 mm	40 mm	27 mm	37 mm
Liner thicknees: front	n/a	15 mm	9 mm	13 mm	8 mm	15 mm
Liner thickness: side	n/a	15 mm	10 mm	12 mm	10 mm	15 mm
Liner thickness: rear	n/a	15 mm	10 mm	20 mm	13 mm	15 mm
Webbing	6-point harness	6-point harness	6-point harnesss	comfort pad	6-point harness	comfort pad
Standard	ANSI Z89.1–2014	ANSI Z89.1–2014	ANSI Z89.1–2014	ANSI Z89.1–2014	EN 12492 (Mountaineering)	n/a (prototype)

∗ Height offset measured from the top of the head and the top of the crown of the helmet shell.

∗∗ Thickness of EPS impact liners was measured at locations corresponding to the crown, front, side and rear impact locations.

### 2.2 Test setup

Helmet testing was conducted at the Helmet Impact Testing (HIT) facility of the Portland Biomechanics Laboratory [14, 29, 30]. Ten helmets of each of the six helmet models were tested in two distinct scenarios to assess impact energy attenuation representative of impacts from falling objects, and representative of falls as tested in more realistic, oblique impacts.

**Figure 2:**
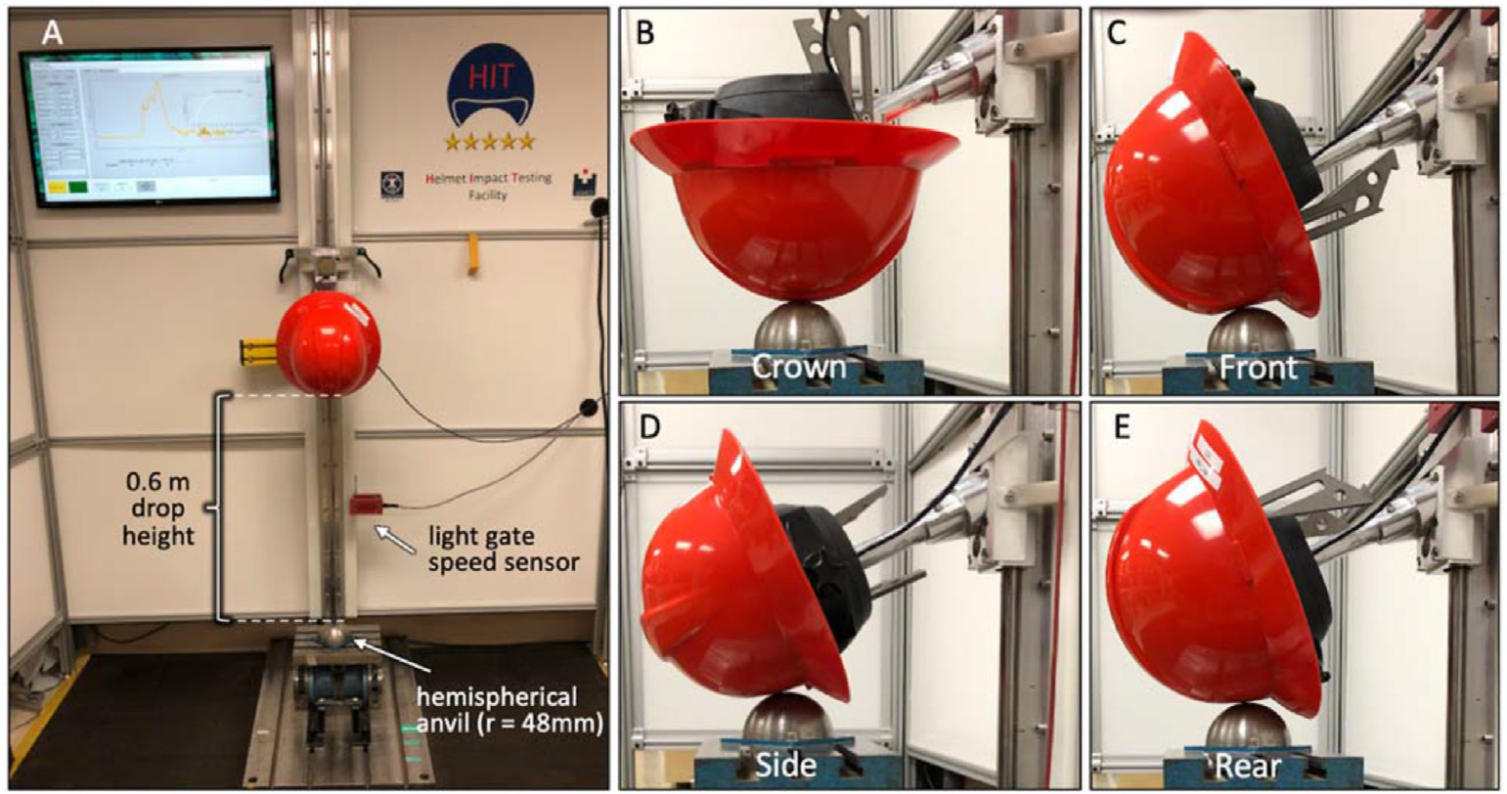
Helmet testing with 31 J impact energy at 3.5 m/s impact speed onto a hemispherical anvil (A), according to Z89.1–2014. Testing was conducted on crown (B), front (C), side (D), and rear (E) locations.

Impacts representative of falling objects were conducted according to ANSI standard Z89.1 for assessment of impact energy attenuation required for Type II helmets (Figure 2). An ISO size J headform with a Shore "D" durometer of 60 (SB070, Cadex Inc., Quebec, Canada) was mounted to the drop assembly of a vertical drop rail. The combined weight of the headform and drop assembly was 5.0 kg. A ball joint inside the headform allowed adjustment of headform orientation for apex, front, side and rear impacts. Since ANSI standard Z89.1 requires that the edge of the hemispherical anvil does not overlap with the Dynamic Test Line (DTL) in front, side, and rear impacts, impact locations were marked to be 48 mm superior to the DTL. Marking of impact locations was performed with a laser level after helmets were firmly seated onto an ISO size J headform and loaded with a 50N static force according to standard ANSI Z89.1–2014. For impact testing, helmeted headforms were subjected to guided freefall from a nominal drop height of 0.6 m to achieve an impact speed of 3.5 m/s, representing an impact energy of 31 J. Impact speed was measured with a timed light gate (#5012 Velocimeter, Cadex Inc., Quebec, CA) located 5 mm above the point of impact. Drop tests were conducted onto a 48 mm radius hemispherical anvil that was rigidly mounted on a solid steel base of 150kg weight. Linear acceleration (*a*_*Z*_) during impact was measured with a linear accelerometer (356B21, PCB, Depew, NY) mounted at the center of gravity of the headform, and oriented to capture acceleration along the vertical z-axis. Five specimens of each helmet model were impacted at 3.5 m/s onto the front side, rear, and crown locations in accordance with ANSI standard Z89.1–2014.

**Figure 3:**
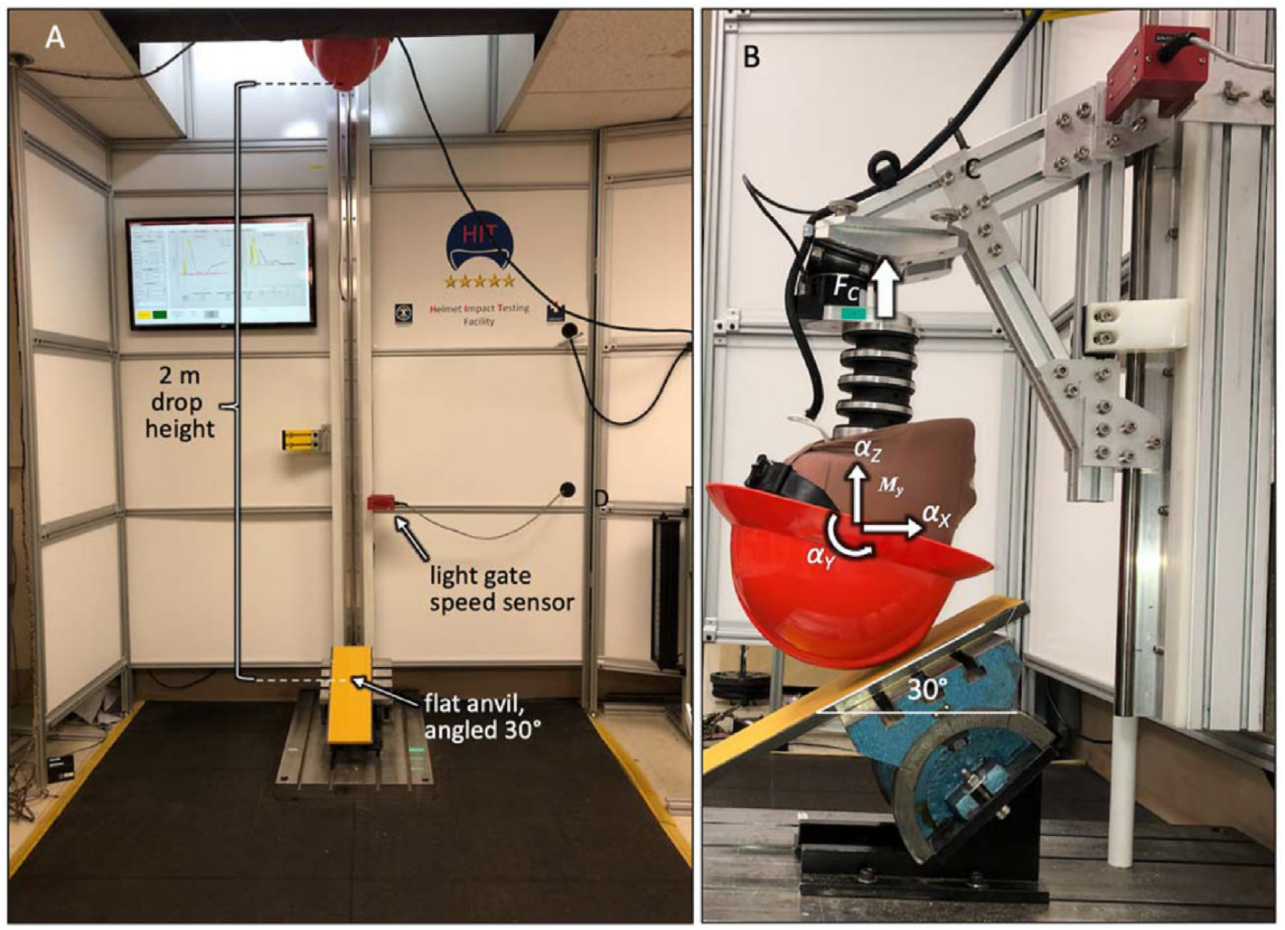
A) Helmet testing with 275 J impact energy at 6.2 m/s impact speed onto a 30° tilted anvil. B) An instrumented Hybrid III head and neck surrogate captured headform acceleration and neck loading.

Impacts representative of falls were tested according to an established protocol of the HIT facility that has been successfully used to determine differences in impact performance of bicycle and snow sport helmets (Figure 3A) [14, 15, 29, 30]. This protocol employed a Hybrid III 50^th^ percentile male anthropomorphic head and neck surrogate (78051-336, Humanetic Innovative Solutions, Plymouth, MI) that was connected to a vertical drop tower rail (Figure 3B). The weight of the drop assembly was 14.3 kg, including the Hybrid III head and neck surrogate and its structural connection to the drop rail. A flat anvil was positioned at a 30° angle relative to the horizontal plane to induce an oblique impact in response to a vertical drop. This 30° angle was selected to match previously published studies of angular acceleration in oblique head impacts [30, 31, 32, 33]. Linear and rotational accelerations of the headform were captured with a six-degrees-of-freedom sensor package (6DX Pro, DTS Inc., Seal Beach, CA) containing three linear accelerometers and three angular rate sensors. This miniature sensor package was mounted at the center of gravity of the Hybrid III head. The resultant linear acceleration *a*_*R*_ was calculated from the three linear acceleration components. The resultant rotational acceleration *α*_*R*_ of the headform was calculated by differentiation of the three angular rate signals. Additionally, the base of the surrogate neck was instrumented with a load cell (IF-203, FTSS Inc.) that measured neck compression (*F*_*C*_). Since the silicone skin surrogate of the Hybrid III headform has over twice the surface friction coefficient of the human head [34], a nylon stocking was fitted over the Hybrid III headform to reduce surface friction. This approach was adopted from prior studies that utilized the Hybrid III headform in helmeted drop tests [13, 14, 15, 29, 35, 36, 37]. Helmets were properly fitted to the headform with their original fit system in accordance with the manufacturers’ fit recommendations. Before each test, new 80 grit sandpaper was applied to the anvil surface [38]. Five specimens of each of the six helmet models were impacted with an impact speed of 6.2 m/s and impact energy of 275 J onto an impact location at the helmet front. This impact speed corresponds to a drop height of 1.96 m and is used by safety standard §1203 of the US Consumer Product Safety Commission (CPSC, 1998) and ASTM standard F1447-12 for impact simulation of bicycle helmets onto a flat anvil [19, 20]. All helmet tests were conducted under ambient conditions, defined according to the ANSI Z89.1 standard to be 23 ± 3 °C, and 50 ± 5% relative humidity [39].

### 2.3 Data acquisition and analysis

For all impact tests, impact kinematics data were captured at a sampling rate of 20 kHz in a data acquisition system (PCI-6221, National Instruments, Austin, TX). For impacts representing falls, linear acceleration signals *a*_*x,*_ *a*_*y,*_ and *a*_*z*_ were low-pass filtered at Channel Frequency Class (CFC) 1000 [40] before calculation of the resultant liner acceleration *a*_*R*_. Rotational acceleration histories *α*_*x*_, *α*_*y*_, and *α*_*z*_ were calculated by differentiation of rotational velocity signals *ω*_*x*_, *ω*_*y*_, and *ω*_*z*_, and were used to calculate the resultant rotational acceleration *α*_*R*_*.*

Three distinct injury probabilities were derived from head kinematic measurements. First, the probability of head injury was assessed by the Head Injury Criterion (HIC-15) of the National Highway Traffic Safety Administration (NHTSA), based on 15 ms linear acceleration histories. Second, the probability of sustaining brain injury with an Abbreviated Injury Score of 2 (AIS2) was calculated from peak rotational velocity according to Eq. (1):

(1) $$P\;\left(AIS2\right)=1-e^{-{(\frac{BrIC}{0.567})}^{2.84}}$$

whereby BrIC = *ω*_*y, peak*_*/56.45, and ω*_*y, peak*_ *is the peak rotational velocity around a transverse axis in response to a frontal impact.* [41].

Finally, the Combined Probability (CP) of concussion was derived from peak linear acceleration *a*_*R*_ and peak rotational acceleration *α*_*R*_ according to Eq. (2) [42]. This injury metrics was derived from over 63,000 sports impacts recorded from instrumented football players, and was validated by impact reconstructions of 58 impacts, including 25 concussions, using Hybrid III test dummies [42].

(2) $$\text{CP}=\frac{1}{1+e^{-\left(-10.2+0.0433a+0.000873\alpha -0.00000092a\alpha \right)}}$$

For statistical analysis, headform kinematics, neck loading *F*_*C*_, and the three injury probability metrics of the 5 helmet groups with advanced designs were compared individually for each outcome parameter to baseline results obtained for the standard Type I hardhat. Two-sided Student's t-tests with Bonferroni correction were used to account for multiple comparisons. A level of α = 0.05 was used to detect statistical significance.

## 3 Results

For impact testing representative of falling objects according to ANSI standard Z89.1, there was no significant difference in the average impact speed and energy between groups. Recorded average speeds ranged from 3.51 ± 0.02 m/s to 3.52 ± 0.02 m/s, and corresponding impact energies ranged from 30.8 ± 0.3 J to 31.0 ± 0.3 J.

**Figure 4:**
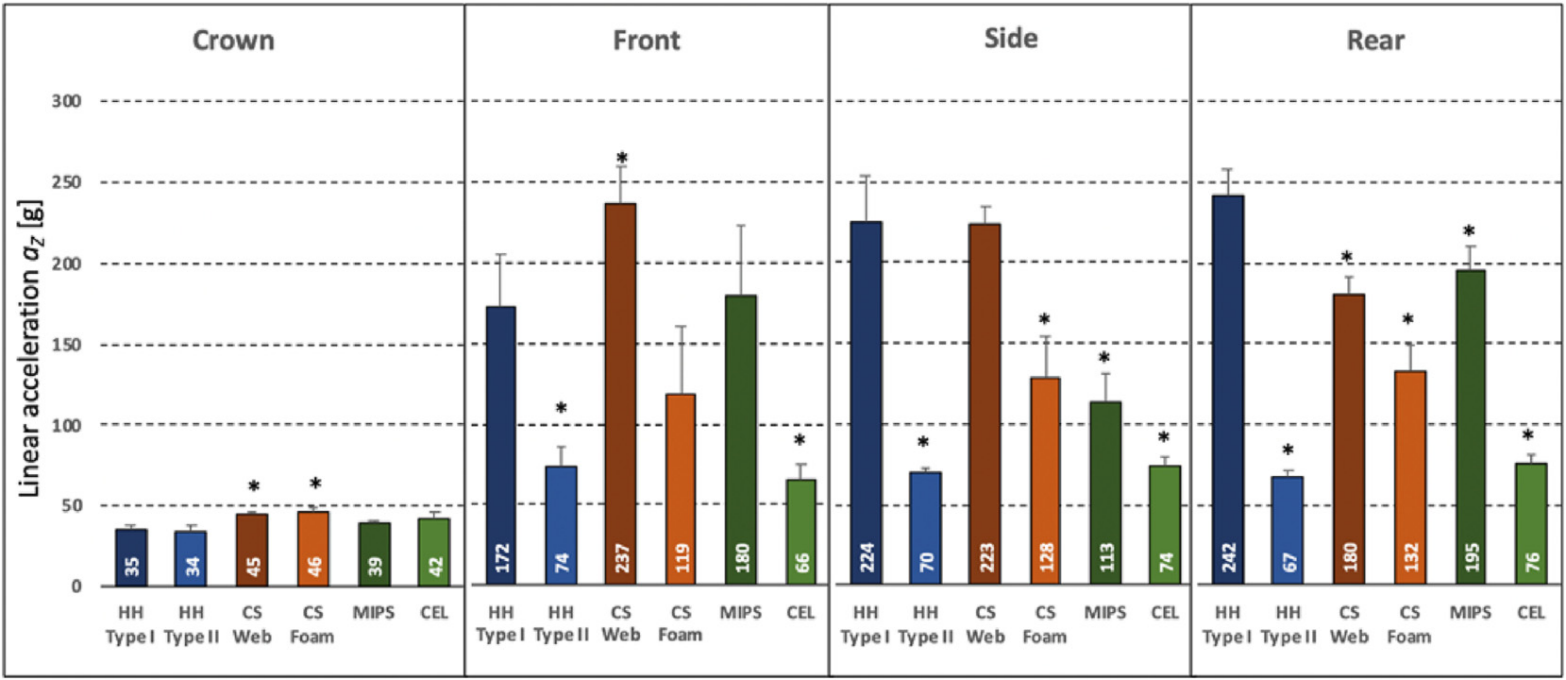
Head acceleration resulting from a 31 J impact at 3.5 m/s to the crown, front, side and rear of the helmet, as specified in ANSI standard Z89.1. Asterisks denote significant differences compared to HH Type I baseline helmets.

Crown impacts induced peak linear accelerations *a*_*Z*_ of less than 50 g in all of the six helmet models, ranging from 35 ± 4 g (HH Type II) to 46 ± 3 g (CS Foam) (Figure 4). Compared to crown impacts, front, side, and rear impacts caused several-fold higher accelerations of 172 ± 4 g, 224 ± 28 g, and 242 ± 15 g, respectively, in the HH Type I group. Compared to the baseline HH Type I helmets, only HH Type II and CEL helmets consistently yielded significantly lower accelerations in front, side, and rear impacts. For HH Type II and CEL helmets, linear acceleration remained below 80g in all impact scenarios. CS Web helmets had a significantly higher acceleration of 237 ± 23 g in front impacts compared to the baseline HH type I helmet. CS Foam helmets exhibited their highest acceleration of 132 ± 17 g in rear impacts. Similarly, MIPS helmets exhibited their highest acceleration of 195 ± 15 g in rear impacts. Test samples in each of the HH Type I, ES Web, ES Foam, and MIPS groups exceeded the 150 g threshold required to meet the Type II off-crown impact rating of ANSI standard Z89.1.

For impact testing representative of falls using a hybrid III head and neck surrogate, there was no significant difference in the average impact speed and energy between groups. Recorded average impact speeds ranged from 6.18 ± 0.02 m/s to 6.22 ± 0.02 m/s, and corresponding impact energies ranged from 273 ± 2 J to 275 ± 2 J.

**Figure 5:**
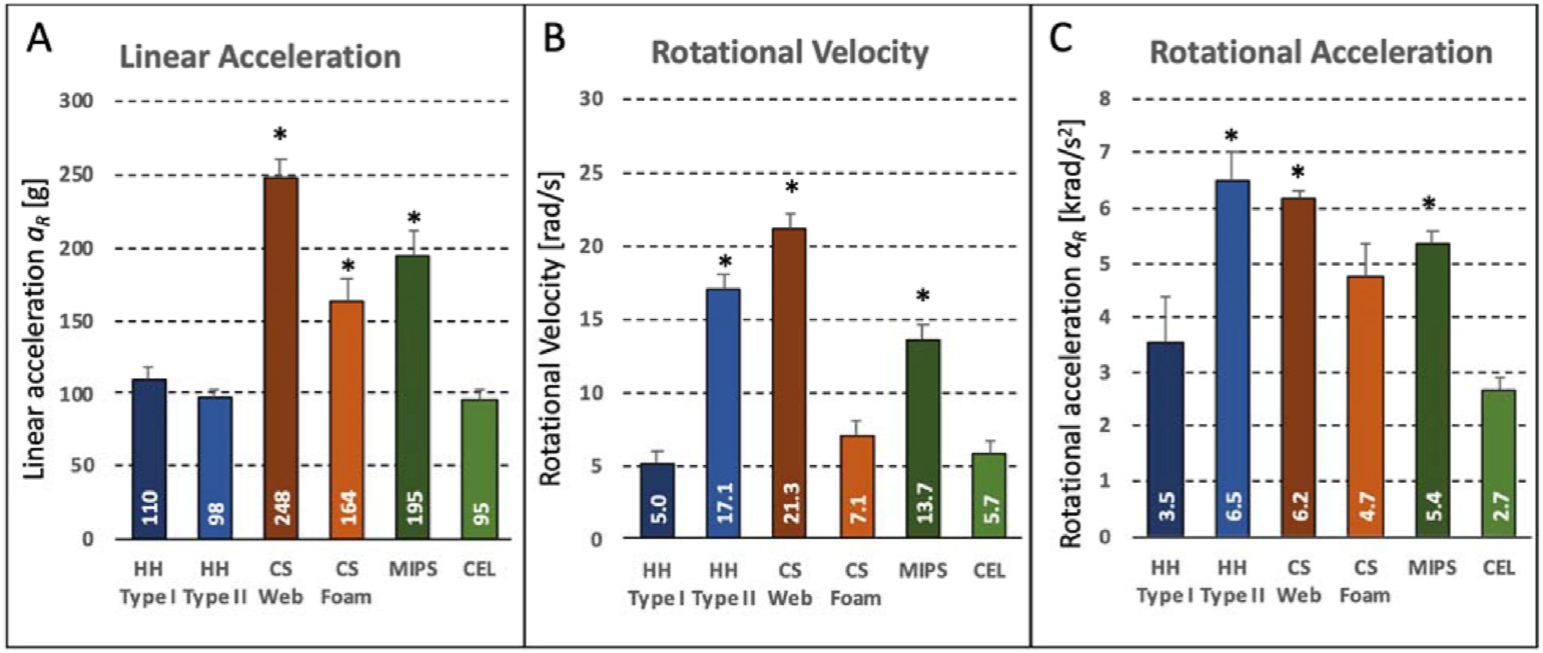
Peak linear acceleration (A), rotational velocity (B), and rotational acceleration (C) of the head resulting from a 275 J impact at 6.2 m/s onto a 30° inclined anvil, using a Hybrid III head and neck surrogate. Asterisks denote significant differences compared to HH Type I baseline helmets.

Resultant peak linear acceleration *a*_*R*_ ranged from 95 ± 7 g (CEL group) to 248 ± 12g (CS Web group) (Figure 5A). Compared to the baseline group (HH Type 1, 110±9g), linear acceleration was significantly elevated in the CS Web group, CS foam group (164 ± 15g), and MIPS group (195 ± 16g).

Resultant peak rotational velocity *ω*_*R*_ ranged from 5.0 ± 2.7 rad/s (HH Type 1 group) to 21.3 ± 0.8 rad/s (CS Web group) (Figure 5B). Compared to the baseline group (HH Type 1), peak rotational velocity was significantly elevated in the HH Type II group (17.1 ± 4.1 rad/s), CS Web group (21.3 ± 0.8 rad/s), and MIPS group (13.7 ± 1.6 rad/s).

Resultant peak rotational acceleration *α*_*R*_ ranged from 2.7 ± 0.2 krad/s^2^ (CEL group) to 6.5 ± 0.2 krad/s^2^ (CS Web group) (Figure 5C). Compared to the baseline group (HH Type 1, 3.5 ± 0.8 krad/s^2^), rotational acceleration was significantly elevated in the HH Type II group, CS Web group (6.1 ± 0.2 krad/s^2^), and MIPS group (5.3 ± 0.2 krad/s^2^).

**Figure 6:**
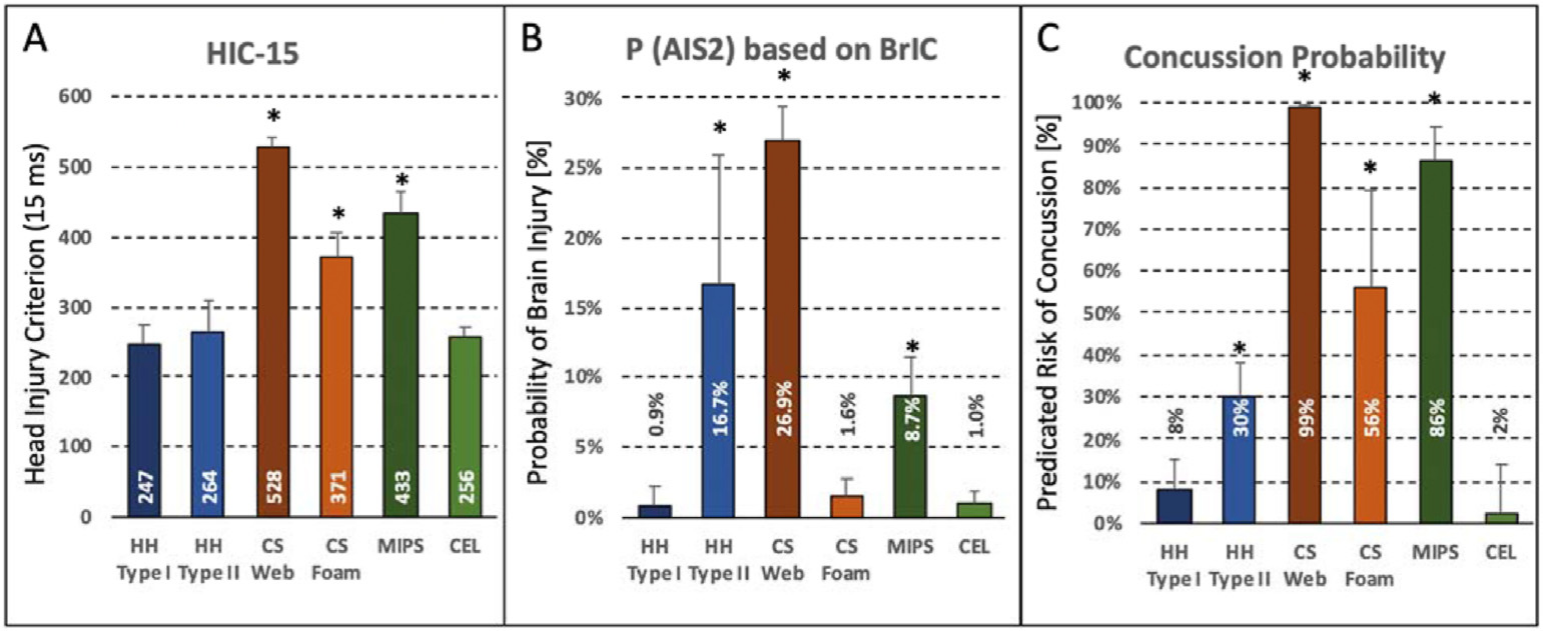
Predicted probability of sustaining a head injury in terms of HIC-15 (A), AIS 2 brain injury (B), and a concussion (C) from a 275 J impact at 6.2 m/s onto a 30° inclined anvil. Asterisks denote significant differences compared to HH Type I baseline helmets.

The probability of head injury, expressed in terms of HIC-15, ranged from 247 ± 29 (HH Type 1 group) to 528 ± 15 (CS Web group) (Figure 6A). Compared to the baseline group (HH Type 1), HIC-15 was significantly elevated in the CS Web group, CS foam group (371 ± 35), and MIPS group (433 ± 32).

The probability P(AIS 2) of sustaining brain injury ranged from 0.9 ± 1.4% (HH Type 1 group) to 26.9 ± 2.5 (CS Web group) (Figure 6B). Compared to the baseline group (HH Type 1), P(AIS 2) was significantly elevated in the CS Web group, HH Type II group (16.7 ± 9.2%), and MIPS group (8.7 ± 2.8%).

The predicated risk of concussion, expressed in the Combined Probability (CP) of concussion, ranged from 2 ± 1% (Cel group) to 99 ± 1% (CS Web group) (Figure 6C). Compared to the baseline group (HH Type 1, 8 ± 7%), the predicted concussion risk was significantly elevated in the HH Type II group (30 ± 8%), CS Web group, CS foam group (56 ± 23%), and MIPS group (86 ± 8%).

**Figure 7:**
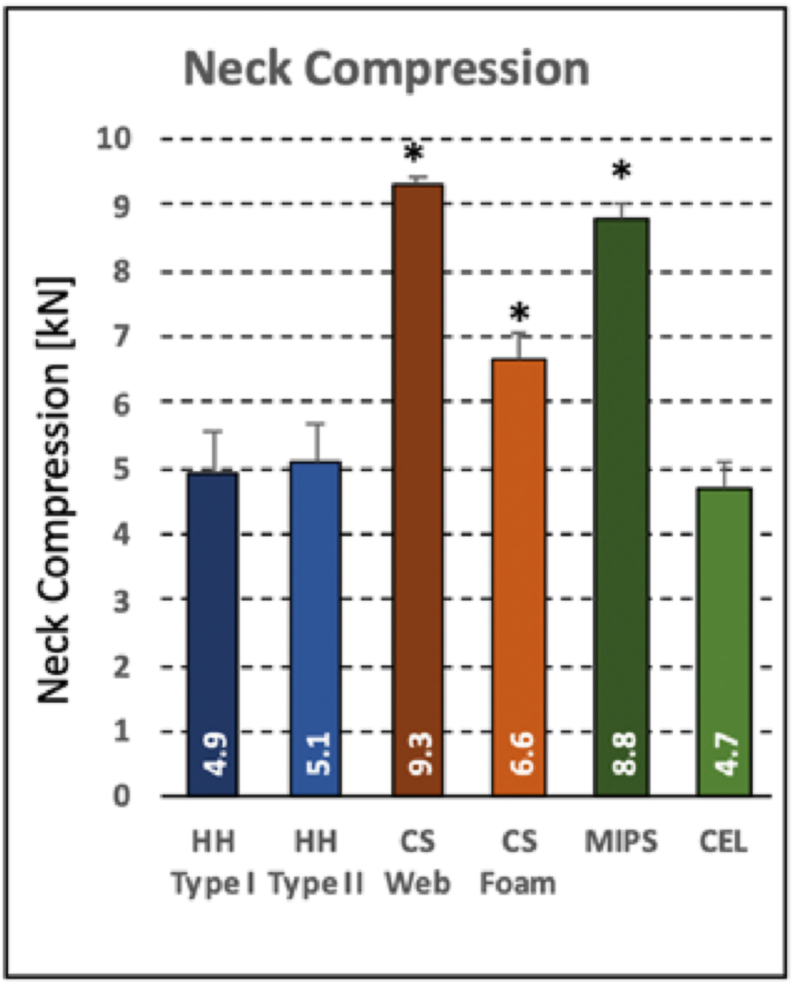
Neck loading during a 275 J impact at 6.2 m/s onto a 30° inclined anvil. Asterisks denote significant differences compared to HH Type I baseline helmets.

The peak neck compression force *F*_*C*_ ranged from 4.7 ± 0.4 kN (Cel group) to 9.3 ± 0.1 kN (CS Web group) (Figure 7). Compared to the baseline group (HH Type 1, 4.9 ± 0.6 kN), neck loaiding was significantly elevated in the CS Web group, CS foam group (6.6 ± 0.4kN) and MIPS group (8.8 ± 0.2kN).

## 4 Discussion

Results of the impact energy attenuation tests according to ANSI standard Z89.1–2014 demonstrated that the design of safety helmets is optimized to meet minimum performance requirements but falls short of providing optimized protection from real-world impacts. All six helmets attenuated the linear acceleration resulting from crown impacts. However, only the Type II rated helmet and the CEL prototype provided similar levels of energy attenuation for lateral impacts which occur three times more frequently than impacts to the crown [11, 18]. In the absence of a mechanism for energy attenuation from lateral impacts, HH Type I helmets exhibited a 5- to 7-fold increase in head acceleration from lateral impacts compared to crown impacts.

In 1980, Koch et al. demonstrated that lateral impacts of only 3.5J energy transmitted forces of over 950 N, concluding that it is surprising that helmet have not been designed for higher protection from lateral impacts [43]. A 1987 study by Gilchrist and Mills similarly concluded that while industrial safety helmets performed adequately in crown impacts they were practically useless against side, front and rear impacts, making a redesign necessary [11]. According to Canadian standard Z94.1–15 for protective headwear, Type I crown-only protective headwear has limited use and shall only be considered if it can be demonstrated that no lateral impact hazards exist [44]. Despite the well-established limitation of Type I helmets, most contemporary safety helmets fulfill only the minimum Type I performance requirement, including modern climbing style helmets that are generally perceived to provide advanced protection.

Addition of a simple EPS liner sufficed to greatly improve attenuation of lateral impacts in HH Type II helmets. While CS Web, CS Foam, and MIPS helmets also included EPS liners, they did not yield the same protection required by Type II helmets. This may be attributed to three factors: first, their EPS liners were thickest at the crown, and gradually thinned towards the sides; second, their EPS liners did not extend to the brim, providing only partial EPS coverage of lateral helmet aspects; and third, the density of their EPS liners was lower than that of HH Type II helmets.

Results of impact testing representative of falls evaluated helmet effectiveness at a 9 times higher impact energy of 275J and captured linear and rotational accelerations as well as neck forces. Under this impact condition, there was no significant difference in resultant linear acceleration between the Type I and Type II hardhats. This may be due to the impact location being sufficiently close to the helmet crown, allowing the impact to be absorbed by the 6-point harness without benefiting from the EPS liner present in the HH Type II helmet. Moreover, the flat rather than hemispherical impact surface distributed the impact over a larger area on the helmet shell which furthermore prevented contact between the shell and headform. All three climbing-style helmets (CS Web, CS Foam, MIPS) exhibited a significantly higher linear acceleration than Type I hardhats. This may be attributed to a higher shell stiffness, since the climbing style shells were made from stiffer ABS rather than HDPE, and had thicker crown sections than HH Type I shells. However, further studies with a focused range of design parameters will be required to draw definitive conclusions on the effect of individual shell parameters on helmet performance.

Mitigation of peak rotational velocity and acceleration of the head greatly varied between helmet types. The simple harness suspension of Type I hardhats yielded a considerably low rotational acceleration of 3.5 rad/s^2^, as it readily provides a rotational suspension. This result closely correlates to a previous study, reporting a 3.5 rad/s^2^ rotational head acceleration for a 244 J crown impact onto a Type I hardhat that was mounted on a hybrid III head and neck surrogate [45]. Type II helmets with the added EPS liner exhibited an 86% higher rotational acceleration that Type I hard hats. This may be attributed to the added constraint of the head inside the helmet. A significantly elevated rotational acceleration was also recorded for the two climbing-style helmets with harness suspension and EPS liner, CS Web (6.2 krad/s^2^), and MIPS (5.4 krad/s^2^). These results correlate to a prior study of climbing-style safety helmets, reporting peak rotational head accelerations of 5.9–6.0 krad/s^2^ for 98 J lateral impacts of a free-falling helmeted headform onto a flat impact surface [46]. Most interestingly, MIPS helmets had a significantly increased rotational acceleration compared to Type I hardhats despite their dedicated MIPS low friction layer. Other studies have shown that MIPS technology can consistently reduce rotational velocity and acceleration [14, 16, 17, 47]. Abayazid et al. evaluated 15 different bicycle helmets with MIPS technology and one helmet with WaveCel technology in frontal impacts at 6.3 m/s onto a 45° anvil in comparison to conventional helmets without rotation damping technology [47]. MIPS and WaveCel helmets similarly reduced peak rotational velocity on average by 19.5% (range 2.5–47%) and 20%, respectively, compared to conventional helmets. In the present study, MIPS helmets and CEL helmets yielded peak rotational velocities of 13.7 rad/s and 5.7 rad/s, respectively. Given the wide range of rotational velocity reductions reported for MIPS helmets by Abayazid et al., the benefits of MIPS technology may rely on proper integration of the MIPS layer into the helmet and the helmet design itself. Therefore, rotation-damping technologies require physical validation to quantify the actual benefits in a particular helmet design. CEL protypes yielded the lowest rotational acceleration and concussion probability, even in absence of a formal design integration into a helmet product. Most recently, Chung et al. compared bicycle helmets with and without a WaveCel liner in the same bicycle helmet design [48]. In frontal impacts at 4.8 m/s onto a 30° anvil, WaveCel helmets yielded a 29% lower rotational velocity, a 17% lower rotational acceleration, and P (AIS 2) decreased by 22% compared to the same helmet design with a full-thickness EPS liner. In the present study, CEL prototypes yielded a 23% lower rotational acceleration than the baseline HH Type 1 helmet, but a comparable rotational velocity and P (AIS 2) value. While this demonstrated the rotation damping efficacy of a simple strap suspension, HH Type 1 helmets were highly deficient in mitigating lateral impacts to the front, side and rear according to the ANSI standard test.

The relevance of neck compression results may best be interpreted in the context of neck loading during sports activities that present a minimal risk of injury, and those neck loads that cause neck injury. Funk et al. determined neck loading in response to heading a soccer ball at 11.5 m/s for 20 human volunteers [49]. They reported average neck compression of 414 N, which is approximately one order of magnitude smaller than neck compression reported for Type I hardhats in the present study. For injurious neck loading, cervical quadriplegia from real-life head-first impacts in athletes was associated with neck compression forces in the range of 3.6–8.1 kN [50]. Biomechanical studies induced compression fractures of cadaveric neck specimens in response to 7.5 kN compressive impact loading [51]. In human cadaveric head and neck specimens, bony and soft tissue injuries resulted from compressive impact forces ranging from 1.6 - 6.2 kN [52]. In the present study, neck compression forces for climbing-style helmets (CS Web, CS Foam, MIPS) ranged from 6.6 kN to 9.3 kN, and were significantly higher than for Type I hardhats (4.9 kN). A prior study reported neck compression of 6.9 kN for a 244 J crown impact onto a Type I hard [45]. Since the magnitude of these neck loads correspond to the injurious neck loading range, mitigation of neck loading should be considered for optimization of helmet designs.

Results of this study described relative performance differences between six safety helmet designs, tested in two distinct impact scenarios that represent impact from falling objects and falls. Results are therefore limited to these specific study parameters and may not be extrapolated outside the tested parameter range. The test setup and parameters were selected to align as much as possible with established test standards and precedence from published studies to facilitate reproduction of the test setup in other test facilities. In absence of reports on impact angles for falls leading to work-related brain injury, a 30° impact anvil was selected to represents the lower, shallow end of commonly used anvil angles for oblique impact testing. Impact testing by guided free-fall onto an angled anvil [30, 31, 53, 54, 55] was chosen over vertical drops onto a laterally translating impact surface [56, 57, 58] or pendulum impact tests [59, 60] for its greater simplicity and high reproducibility [56]. The Hybrid III 50^th^ percentile male anthropomorphic head was chosen, since it readily allows for sensor integration and Hybrid III neck attachment. Controversy remains if oblique impact testing should be conducted with a Hybrid III neck surrogate [13, 14, 15, 25, 29, 30, 35, 36, 37, 53, 57, 59] or with an unconstrained headform [31, 54, 55, 58]. Fahlstedt et al. used finite element analysis to compare head-first impacts of head-only models and full-body models [28]. Inclusion of the full body influenced head linear and rotational accelerations during the first 15 ms by only 5–8%. Moreover, Feist et al. showed that rotational peak velocity is comparable in full-body and head-only impacts [61]. These two finite element studies support oblique impact simulation without a Hybrid III surrogate. There are also biofidelity limitations of the Hybrid III neck surrogate, which was developed for automotive crash testing and not for sports impacts [62]. It was validated in flexion and extension, but has been shown to be overly stiff in lateral bending for which reason only frontal impacts were tested in the present study [63]. Moreover, the Hybrid III neck surrogate has been criticized for its high-stiffness response during axial loading [64] and for its non-biofidelic storage of elastic energy that can confound the late recoil phase of the impact [48]. In the presents study, a mid-sagittal, frontal impact location was chosen to achieve predominantly flexion and extension motion for which the Hybrid III neck has been validated, and to match the impact scenarios in previously published studies [30, 31, 33, 56, 58]. Despite the biofidelic limitations of the Hybrid III neck, other studies argue that its presence incorporates in part an effective body mass which can affect head kinematics and helmet crushing [65, 66]. Ghajari reported that the presence of a body affects rotational head acceleration by up to 40%. It furthermore increases impact energy and hence the crushing distance of a helmet liner, which is a key parameter in designing helmets [65]. The question remains whether the limitations of the Hybrid III neck outweigh the biofidelic limitations of foregoing the use of a torso mass and neck during helmet testing [48].

In addition to limitations due to simplified simulation of real-world impacts under reproducible laboratory conditions, further limitations must be considered when predicting brain injury risk from impact kinematics data. Headform kinematics were analyzed to calculate the combined probability (CP) of concussion and the probability P(AIS 2) of sustaining brain injury. However, prediction of the concussion and brain injury risk depends on the accuracy of injury risk curves that have been reconstructed from a limited number of real-world injury data to estimate brain tolerance limits. Moreover, these injury risk curves are highly non-linear, for which reason a relatively small difference in peak rotational velocity can translate into a large difference in injury probability [53]. The uncertainty in defining brain tolerance limits combined with the non-linear nature of injury risk curves necessarily limits the accuracy in predicting an absolute probability of brain injury. However, relative differences in brain injury probability between helmet technologies should provide a meaningful comparison since the helmet designs were tested under defined and reproducible impact conditions. Nevertheless, future studies will be required to expand the parameter range of impact conditions, and to include additional helmet designs.

## 5 Conclusions

Industrial safety helmets are the most effective intervention to prevent head injury. It therefore remains imperative that their impact energy attenuation is optimized not to a minimum performance criterion of a standard but to address realistic injury scenarios, including lateral impacts and falls.

Today's advanced helmet designs do not necessarily deliver improved mitigation of linear and rotational impact forces and may not provide a higher level of injury protection from impacts and falls than traditional hardhat helmets. Results of this study quantified the deficient mitigation of lateral impacts inherent to Type I helmets. Since lateral impacts are more frequent than crown impacts, the use of Type II helmets with lateral impact protection seems strongly advisable. However, this study also demonstrated that the Type II hardhat significantly increased the rotational acceleration, rotational velocity and resulting concussion risk and P(AIS 2) brain injury probability compared to the Type I hardhat, likely due to the added head constraint from the side protection liner.

While climbing-style safety helmets are perceived and marketed as a safer alternative to traditional hardhats, results of this study do not support this notion. All three climbing-style safety helmets offered less protection from side impacts than the Type II hardhat, and exhibited a great concussion risk and neck loading than the Type I hardhat. As such, helmet performance is not linked to a particular helmet style, and advanced helmet designs do not necessarily deliver improved injury protection from falling objects and falls. CEL helmets with a dedicated rotation-damping technology also provided Type II level side protection, demonstrating the potential to improve protection from side impacts and falls. The observed differences in efficacy between helmet technologies emphasize the need for helmet testing and for continued research and development to further improve the protective performance of industrial safety helmets.

## Declarations

### Author contribution statement

Michael Bottlang, Stanley Tsai, Steven Madey: Conceived and designed the experiments; Analyzed and interpreted the data; Wrote the paper.

Gina DiGiacomo: Conceived and designed the experiments; Performed the experiments; Wrote the paper.

### Funding statement

Michael Bottlang was supported by Legacy Research Foundation (11074).

### Data availability statement

Data will be made available on request.

### Declaration of interest’s statement

The authors declare the following conflict of interests: Some of the authors (MB, SMM) have previously received NIH awards for research on CEL technology described in this manuscript, are co-inventors of the CEL technology, and have a financial interest in the company that owns this technology. None of the authors received any money or in-kind contribution for this work.

### Additional information

No additional information is available for this paper.
